# Integrative oncology and complementary medicine cancer services in Australia: findings from a national cross-sectional survey

**DOI:** 10.1186/s12906-018-2357-8

**Published:** 2018-10-29

**Authors:** Caroline A. Smith, Jennifer Hunter, Geoff P. Delaney, Jane M. Ussher, Kate Templeman, Suzanne Grant, Eleanor Oyston

**Affiliations:** 1 NICM Health Research Institute, Western Sydney University, Westmead campus, Locked Bag 1797, Penrith, NSW 2751 Australia; 2 Menzies Centre for Health Policy, School of Public Health, Sydney Medical School, The University of Sydney, Sydney, NSW Australia; 3 Translational Health Research Institute, School of Medicine, Western Sydney University, Campbelltown, NSW Australia; 4 South-Western Sydney Clinical School, Faculty of Medicine, University of New South Wales, Kensington, NSW Australia; 5 Cancer Services, South Western Sydney Local Health District, Liverpool, NSW Australia; 6 Ingham Institute of Applied Medical Research, Liverpool, NSW Australia; 7 Oncology Massage Limited, PO Box 109, Deakin West, ACT 2600 Australia

**Keywords:** Cancer, Supportive care, Complementary medicine, Integrative oncology, Integrative medicine

## Abstract

**Background:**

Individuals living with and beyond a cancer diagnosis are increasingly using complementary therapies and medicines (CM) to enhance the effectiveness of cancer treatment, manage treatment-related side effects, improve quality-of-life, and promote self-efficacy. In response to the increasing use and demand for CM by cancer patients, interest in the implementation of Integrative Oncology (IO) services that provide CM alongside conventional cancer care in Australia and abroad has developed. The extent that cancer services in Australia are integrating CM is uncertain. Thus, the aim of this study was to identify IO services in Australia and explore barriers and facilitators to IO service provision.

**Methods:**

A national, cross-sectional survey of healthcare organisations was conducted in 2016. Organisations in the public and private sectors, including not-for-profit organisations that provided cancer care in hospital or community setting, were included.

**Results:**

A response rate of 93.2% was achieved (*n* = 275/295). Seventy-one organisations (25.8%) across all states/territories, except the Northern Territory, offered IO albeit in a limited amount by many. Most common IO services included massage, psychological-wellbeing, and movement modalities in hospital outpatient or inpatient settings. There were only a few instances where biological-based complementary medicine (CM) therapies were prescribed. Funding was often mixed, including patient contributions, philanthropy, funding by the organisation, and volunteer practitioners.

Of the 204 non-IO providers, 80.9% had never provided any IO service. Overwhelmingly, the most common barrier to IO was a lack of funding, followed by uncertainty about patient demand, choice of services, and establishing such services. Less-common barriers were a lack of evidence, and support from oncologists or management. More funding, education and training, and building the evidence-base for CM were the most commonly suggested solutions.

**Conclusion:**

IO is increasingly being provided in Australia, although service provision remains limited or non-existent in many areas. Mismatches appear to exist between low IO service provision, CM evidence, and high CM use by cancer patients. Greater strategic planning and policy guidance is indicated to ensure the appropriate provision of, and equitable access to IO services for all Australian cancer survivors.

**Electronic supplementary material:**

The online version of this article (10.1186/s12906-018-2357-8) contains supplementary material, which is available to authorized users.

## Background

Individuals living with and beyond a cancer diagnosis (hereafter referred to as cancer survivors) in Australia, are increasingly using complementary medicine (CM) [1] and some cancer services are providing integrative oncology (IO) services [2, 3]. Integrative oncology (IO) is described as: *“a patient-centred, evidence-informed field of cancer care that utilizes mind and body practices, natural products, and/or lifestyle modifications alongside conventional cancer treatments. IO aims to optimize health, quality of life, across the cancer care continuum and to empower people to prevent cancer and become active participants before, during, and beyond cancer treatment”* [4]*.*

The prevalence of CM use by cancer survivors in Australia has risen from 22% in 1996 [5], to 65% in 2008 [6], with an estimated period prevalence rate between 1985 and 2009 of 43% (95% CI: 19–67%) [1]. The most commonly used CM interventions include biological-based therapies (such as nutritional supplements, special diet and foods, and traditional herbal medicines) followed by non-biologically-based therapies (such as prayer/spiritual practices, meditation/imagery, massage, yoga, acupuncture, Tai Chi/Qigong, and relaxation) [6]. CM is mostly used by cancer survivors as an adjuvant rather than an alternative to their conventional cancer treatment. Reasons for use include desire to augment the effectiveness of treatment, manage treatment-related side effects, improve quality-of-life, and promote self-efficacy [7, 8].

Whilst the research reporting CM use and the experiences of cancer survivors in Australia continues to grow [9–13], little is known about its integration with other cancer services. Only two studies have explored this issue, and the results from both surveys were limited by small sample sizes, restricted inclusion criteria, and suboptimal response rates [2, 3]. Questions remain about the current provision of IO services, the types of CM therapies that are being integrated, the healthcare settings in which they are provided, how they are funded, and key determinants influencing the provision of such services.

## Methods

The aim of this study was to examine current IO service provision in Australia and explore barriers and facilitators to service delivery. A cross-sectional survey of Australian healthcare organisations with cancer services was conducted throughout 2016. The sample was obtained through extensive search strategies to identify all cancer services from both the public and private sectors, including not-for-profit organisations that provided cancer care in either a hospital or community setting. A shortlist of potentially eligible organisations was generated from searching public and private hospital databases and organisations that were located in community settings [14–16]. To ensure potential services were not missed, volunteers from each State who were familiar with the cancer services in their region were given specific instructions for conducting Internet searches on Google and Bing search engines. In addition, further services and sites were identified through conversations with industry experts from peak organisations (e.g. Cancer Nurses Society of Australia, Clinical Oncology Society of Australia, Cancer Council Australia), cancer care networks (e.g. Integrated Cancer Services Managers Group), collaborative groups (e.g. Complementary and Integrative Therapies Group, Western Australian Clinical Oncology Group), and managers and survey participants who provided information about affiliated sites, and/or other locations.

Excluded from the survey were small businesses with specialist consultation rooms only; palliative care services and hospices that were not part of an organisation with cancer services; and organisations that only provided information, support groups, counselling or ad-hoc retreats for cancer survivors. For those services meeting the eligibility criteria, the research officer made contact with organisation volunteers and presented an invitation to participate. Each participating organisation nominated an appropriate staff member to answer the survey. Written, informed consent was obtained from each respondent.

A 52-item questionnaire was designed and pilot tested Additional file 1). Content and questions were based on a NSW survey instrument of CM practices and policies in cancer services [3] and a Scottish scoping study of OM services [17] The online and paper versions of the questionnaire were pilot-tested with staff working in a local cancer service that provided IO and modified accordingly. On-line or paper versions of the questionnaire were available. The online version was administered through SurveyMonkey [18]. Most questions included an option for an open-ended response or comments.

The following broad definitions were provided at the beginning of the questionnaire and are jointly referred to hereafter as IO:

- CM – acupuncture, aromatherapy, chiropractic, herbs and supplements, massage, meditation, music or art therapy, naturopathy, osteopathy, Reiki, relaxation, Tai Chi, therapeutic touch, yoga.
- integrative medicine (IM) – healthcare practitioners who combine evidence-based conventional medicine with CM.

A more comprehensive list of CM services was used when inquiring about service provision for different CM categories (see supplementary material).

Every survey was checked to ensure that there was only one response per organisation and that respondents had not inadvertently selected an incorrect response to the skip question about CM service provision. If either occurred, relevant respondents were contacted and asked to amend their responses. In instances where more than one staff answered the survey, the responses from the most senior person were kept. Those respondents who reported that their cancer service was in the planning stages of delivering a CM service were recontacted before closing date to determine if this prior status was still valid.

Descriptive statistics detailing the counts and percentages was the primary statistical method used. Statistical analysis was undertaken using SPSS V24 [19]. Questions requiring inferential statistical analysis were determined a priori using Chi-squared and Fishers exact tests. Statistical significance set at *p* < 0.05. Qualitative data from the open questions were independently coded for content into descriptive categories by authors CS and JH, and analysed using conventional content analysis [20]. Many of the questions were compulsory, and as such, provided a ‘don’t know’ option. Missing data included unanswered questions. A map of the distribution of IO services was generated using The software use for mapping in this project was ArcGis [21].

## Results

A total of 366 healthcare organisations were identified, from which 295 met the inclusion/exclusion criteria. The response rate was 93.2%, with 275 of the eligible organisations participating in the study. Response rates in the Northern Territory and the Australian Capital Territory were significantly lower than other states, at 66.7% and 75.0% respectively (Fisher’s exact, *p* < 0.05). There were no incomplete surveys.

Most of the 275 respondents (55.6%, *n* = 153) reported dual roles in the organisation as both a healthcare professional and administrator/manager. For the remaining, 73 (26.5%) reported their role as a healthcare professional only, and 49 (17.8%) were an administrator/manager only. Of the healthcare professionals, 60.2% (*n* = 136) had a nursing background, and only a few were an oncologist/haematologist (3.1%, *n* = 7).

### Integrative oncology service provision

Seventy-one organisations (25.8%) stated they offered some type of IO service (Table 1). The median duration of service provision was 6 years, ranging from 2 months to 42 years. Some respondents reported incremental service development, reflecting changes in attitudes towards IO, pressure to provide evidence-informed therapies, and responsiveness to patient needs.

**Table 1:** Location and ownership of integrative oncology providers and non-providers

Healthcare organisations with specialised cancer services *n* = 275	IO providers		Non-IO providers		Total	
	n	%	n	%	n	%
Location						
Australian Capital Territory	1	0.4	2	0.7	3	1.1
New South Wales	25	9.1	57	20.7	82	29.8
Northern Territory	0	0.0	2	0.7	2	0.7
Queensland	9	3.3	58	21.1	67	24.4
South Australia	6	2.2	22	8.0	28	10.2
Tasmania	2	0.7	5	1.8	7	2.5
Western Australia	11	4.0	17	6.2	28	10.2
Victoria	17	6.2	41	14.9	58	21.1
Ownership*						
Government	27	9.8	109	39.6	136	49.5
For-profit company	11	4.0	67	24.4	78	28.4
Not-for-profit company	33	12.0	28	10.2	61	22.2
Total	71	25.8	204	74.2	275	100.0

* Χ^2^ (2) = 33.6, *p* < 0.001

**Fig. 1:**
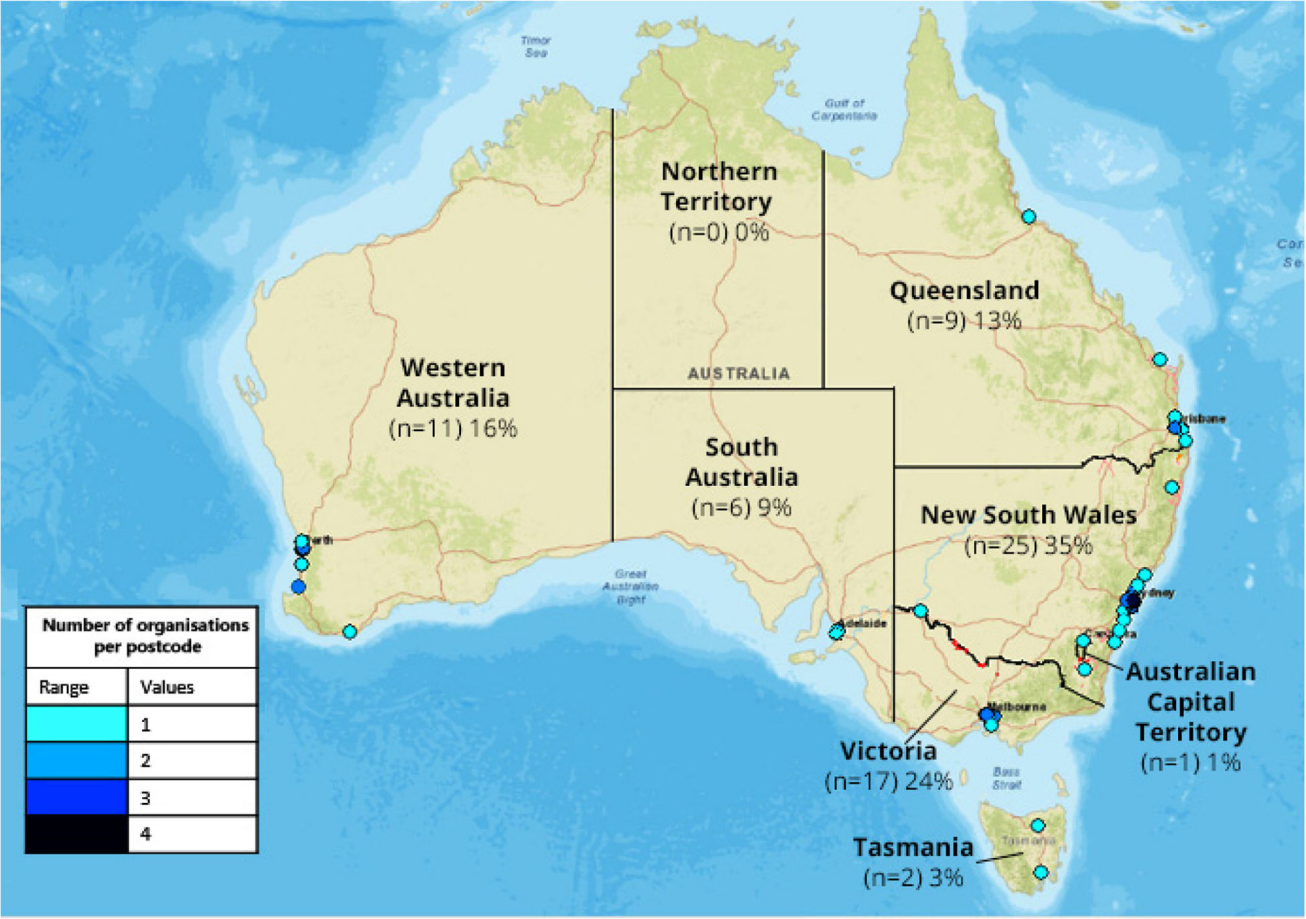
Location of organisations with integrative oncology services

All states, except the Northern Territory, offered IO (Fig. 1). No significant differences between the states were observed (Fisher’s exact, *p* = 0.10). Significant differences, however, were observed between the ownership and the likelihood of providing IO (Fisher’s exact, *p* < .001). IO providers were most likely to be owned by a not-for-profit organisation (46.5%) or were government owned (38.0%), and least likely to be owned by a for-profit organisation (15.5%). In comparison, most non-IO providers were government owned (53.4%), followed by for-profit organisations (32.8%), and not-for-profit companies (13.7%).

IO services were mostly provided in hospital inpatient or outpatient settings (Table 2). In general, the most notable difference between the settings in which IO was provided compared to cancer services was that only 4.2% (*n* = 3/71) of the IO services were provided to patients at home or in residential care compared to 27.6% (*n* = 76/275) for all cancer services. Twenty-five organisations provided some or all of their IO services in a dedicated centre.

**Table 2:** Settings where cancer services are provided

Setting^b^	IO providers IO services^a^		IO providers all cancer services		Non-IO providers all cancer services		All providers (total)	
	n	%	n	%	n	%	n	%
Hospital inpatient	37	13.5	54	19.6	120	43.6	174	63.3
Hospital outpatient / clinic	56	20.4	64	23.3	187	68.0	251	91.3
(Dedicated IO centre)	(25	9.1)						
Community centre / facility	14	5.1	22	8.0	54	19.6	76	27.6
Home / residential care visits	3	1.1	23	8.4	53	19.3	76	27.6
Total	71	25.8	71	25.8	204	74.2	275	100.0

^a^IO services are a sub set of all cancer services ^b^ more than one response allowed

Of the organisations offering IO services the most common IO services were massage (76.1%, *n* = 54/71), psychological wellbeing services (71.8%, *n* = 51/71), and movement modalities (39.4%, *n* = 28/71) (Table 3). The median number of the different categories of IO services (Table 3) was two; 19 organisations provided only one category, and 10 organisations provided four or more. Practitioners generally worked on a part-time basis.

**Table 3:** Integrative oncology (IO) service provision

Service category	Number of organisations		Number of practitioners / organisation		Hours available per week / organisation	
	n	%	Median	range	Median	range
Massage, Touch, or Body Alignment Therapies	54	76.1	2.5	1 to 27	12	2 to 65
Psychological Wellbeing Services	51	71.8	2	1 to 10	7	0.5 to 72
Movement Modalities *(non-CM physiotherapy & exercise physiology excluded)*	28	39.4	2	1 to 20	3	1 to 20
Integrative Medicine (*consultation or advice)*	13	18.3	1	1 to 4	40	24 to 46
Acupuncture *(either medical or Chinese)*	9	12.7	1	1 to 3	6	2 to 24
Other *(naturopath, nutritionist not a dietitian service)*	3	4.2	1	1 to 3	16	6 to 60

Total number of organisations providing IO services *n* = 71

A wide range of massage, touch, and body realignment therapies were offered, with oncology massage (defined as massage provided by a certified oncology massage therapist) being the most prevalent (55.6%, *n* = 30/54). Osteopathy and chiropractic services were not provided by any of the cancer services. The most commonly provided psychological wellbeing services were art therapy, meditation, music therapy, and relaxation. Yoga and Tai Chi were the most frequently reported movement modalities. Ten services reported offering movement modalities delivered by either a physiotherapist (*n* = 7) or exercise physiologist (n = 3); however, nine of these were not included in the final count as the modality was not classified as a CM. Less frequently provided was acupuncture (*n* = 9).

Aside from Western naturopathy, no other holistic traditional healing practices, specifically Chinese herbal medicine, Ayurveda medicine or Indigenous Australian healing practices, were offered. Overwhelmingly, biological-based CM therapies (e.g. herbs, vitamins or minerals) were not provided. There were only four cancer services where such therapies could have been formally prescribed by either an IM doctor or CM practitioner. Formal IM advice from a pharmacist about CM products was available at nine services.

### Qualifications of practitioners

Twenty of the 71 organisations with IO services (28.2%) indicated they had practitioners with dual qualifications (defined as practitioners who held both qualifications as a biomedical trained practitioner and complementary medicine practitioner); however, a similar number (*n* = 21, 29.6%) did not know the answer to this question. Several examples were given that included a nurse certified in oncology massage and another who was also a naturopath; a medical practitioner with acupuncture qualifications; and an occupational therapist who trained as a music therapist.

### Integration of practitioners

Most of the cancer services that provided IO held multidisciplinary team meetings or case conferences (83.1%, *n* = 58) from which just under half (*n* = 28) invited the IO practitioners to participate. Almost an equal number (*n* = 27) indicated that these practitioners were not invited, and four respondents did not know the answer to this question.

### Funding of services

Funding resources were mixed. Organisations used a variety of sources: patient contributions (49.2%, *n* = 35), philanthropic contributions (47.9%, *n* = 34), funding by the organisation (47.8%, n = 34), and volunteer practitioners (42.2%, *n* = 30). Patient contributions were defined as ‘any combination of out-of-pocket costs or rebates from either private health insurance or Medicare’. IM services were the only category of service (Table 3) where none of the IO providers funded the service with philanthropic contributions nor through the help of volunteer practitioners. Not-for-profit organisations were significantly more likely to engage volunteer practitioners (Χ^2^ (2) = 8.9, *p* < 0.05). No significant differences were found between the ownership of the organisation and the likelihood of the IO services being funded by the other three sources.

### Organisations not providing IO

Of the 204 non-IO providers, 80.9% (*n* = 165) had never provided IO services, 7.8% (n = 16) previously provided IO services, and 5.8% (*n* = 12) were planning to provide IO services. Eleven (5.4%) of the non-IO providers commented that the cancer service actively provided information and/or referred patients to nearby IO or CM services. Multiple reasons, including qualitative comments, were given for why the cancer services did not provide IO and barriers to providing IO (Table 4).

**Table 4:** Barriers to providing Integrative oncology (IO) services and potential solutions

Barriers (*n* = 204)	Number	Percent	Potential Solutions (*n* = 130)	Number	Percent
Lack of funding	123	60.3	Funding	59	45.4
Low patient demand / awareness	65	31.9	Staff education / training	30	23.1
Unsure about which IO services to provide	64	31.4	Build the evidence-base	18	13.8
Unsure how to set up an IO service	55	27.1	Help with developing a business model	8	6.2
Lack of support or interest from oncologists	51	25.0	Determine clinical governance	7	5.4
Organisational policy does not allow IO	38	18.6	Change organisational attitudes / culture	7	5.4
Not enough evidence	22	10.8	Ensure sufficient demand for service	5	3.8
Management does not want IO services	16	7.8	Policy support	4	3.1
Other Comments:			More space to provide services	3	2.3
Inadequate resources e.g. time, staff, space	17	8.3			
No champion or organisational interest	8	3.9			
Unsure of patient demand	7	3.4			
Difficulty recruiting CM practitioners	6	2.9			
Patient affordability / high out-of-pocket costs	2	1			

Only non-IO providers were asked these questions. More than one response was allowed

### Integrative oncology barriers and facilitators

Nearly two-thirds (123/188) of respondents identified insufficient funding as the most common reason their organisation did not provide IO services. Similarly, the most common solutions suggested were to address funding dilemmas and establish sustainable business models. How IO services should be funded was more contentious. Some called for *“Medicare funding to support the use of appropriate complementary medicine.”* Others suggested higher rebates from private health insurers. Philanthropy, *“fundraising”* or finding practitioners *“that want to volunteer”* were also proposed. A few respondents from the private health sector considered it was the responsibility of the public service to provide IO services. This view, however, was not always shared by those in the public health sector who, for example, stated that *“given the number of competing demands for resources within a public hospital”*, accessing IO *“would need to be patient/consumer-driven”* and patients could *“seek this if they wish to”* in the community. Other respondents highlighted the challenges with providing affordable, equitable services for their population base.

*“We are currently trying to develop integrative therapies in the centre. Sustainability and cost will always be a factor. We are in a demographically struggling area.”* (Administrator/ Manager and Healthcare professional)

Funding issues were intertwined with other challenges, such as providing value in healthcare and prioritising essential services. For some, IO was considered a non-essential service that would require external funding and evidence to justify its provision.

*“We are too busy complying with accreditation and providing the best possible known treatment services to our patients. I feel we are here to heal people not be airy fairy, there are plenty of places for that. I also feel these complementary treatments belittle what we are trying to achieve. But if they were paid by the Health Funds as inpatient services at great reward I would reconsider this.”* (Administrator/Manager)

*“Difficult, government authorities do not recognise complementary therapies as being essential in supporting cancer patients through cancer treatment and beyond. Grants are great but when the funding runs out the service has to cease in most cases.”* (Administrator/Manager and Healthcare professional)

*“If evidence supports better outcomes for patients when they receive complementary therapy, a business case could be made to include their services”* (Healthcare professional)

Although only a few respondents (10.8%, *n* = 22/204) considered inadequate evidence as a barrier to providing IO services, building a stronger evidence base (13.8%, *n* = 18/130) and educating staff about existing evidence (23.1%, *n* = 30/130) were more commonly suggested as potential solutions (31.3%, *n* = 64/130). For some, establishing an evidence base was paramount.

*“Until there is adequate evidence to support significant objective benefit the other barriers are irrelevant. Oncologist support will only come with evidence.”* (Healthcare professional)

In addition to more research investigating efficacy and cost-effectiveness, respondents also identified uncertainty about patient needs for IO (31.9%, *n* = 65/204). These individuals discussed the importance of obtaining more information about patient demand and needs (2.9%, n = 6/130).

*“Research as to what the patients would like us to consider and how we would fund it.”* (Administrator/Manager and Healthcare professional)

## Discussion

The cross-sectional survey of CM services within healthcare organisations was the largest and most comprehensive of its kind to have been conducted in Australia [2, 3] identifying 295 healthcare organisations with cancer services. Although the provision of IO by these services appears to have doubled over the past 6 years, albeit in a limited capacity by many cancer services, most of the 275 surveyed organisations (74.2%) were yet to provide any type IO service. For the 71 organisations that did, IO services were largely provided in hospital inpatient or outpatient settings, including those with a dedicated IO centre. Access, however, was often limited by availability with services being offered for a limited number of hours per week. Services relied heavily on funding from patients and philanthropy, and the generosity of volunteer CM practitioners.

Challenges with funding IO services, coupled with the need for more guidance on how to establish these services, were considered the greatest obstacles reported by non-IO providers. Insufficient evidence of safety and efficacy, and a lack of support or interest from oncologists or senior management, were other important barriers. These findings are consistent with other research, including a recently published small study of IO organisations in Australia [22], suggesting that barriers to providing IO include challenges with determining an appropriate service model and revenue structure; concerns with clinical governance and legal issues, such as regulations and credentialing; a lack of education about CM; and inadequate evidence about safety and effectiveness of CM [23–27]. Many of these challenges are not unique to IO, and to some extent, reflect the challenges with providing supportive cancer care more generally [28–31], and translating evidence into practice when evidence is established and recommended in clinical guidelines [32]. Indeed, delivering value-based healthcare, along with evidence for effectiveness and cost-effectiveness, places what patients value at the centre of healthcare decision making [33].

Notwithstanding these challenges, the provision of IO services in 2016 are substantially higher at 25.8% (30.5% for New South Wales) compared to earlier estimates of 8.9% for Australia in 2014 [2] (19% for New South Wales in 2009) [3]. Different sampling frames and definitions of IO service provision may explain some of the observed differences. The 2009 New South Wales survey only inquired about CM services for inpatients that were provided by practitioners who were not employees of the hospital [3]. Similarly, the 2014 national survey used only one hospital database to identify organisations with an oncology department, no community-based organisations were included, and it was unclear if inpatient services were included [2]. If community-based organisations and inpatient CM services were excluded from the current analysis, 2016 estimates would remain substantially higher at 20.4% (*n* = 56). Coupled with a six-year mean duration of operation, and a further 12 (4%) organisations that were planning to provide IO, results from the 2016 survey demonstrate substantial ongoing growth of IO service provision in Australia.

Despite this apparent growth, Australian IO service provision appears similar to some comparable countries. In 2009, the estimated number of National Health Service cancer treatment centres in the United Kingdom providing IO ranged from 2.2 per 1 million population in England to 5.0 in Northern Ireland [34]. The comparison rate for Australia is estimated at 2.9 healthcare organisations with an IO service per 1 million population (2016 total population in Australia 24.4million) [35]. A 2013 mapping survey of oncology centres and hospitals in Europe identified 47 of the 99 responding cancer centres provided IO [36]. Response bias, however, may have resulted in an overestimate. Rates for the US, Canada, and New Zealand are yet to be reported, although most of the National Cancer Institute designated comprehensive cancer centres in the US purportedly provide IO [37, 38].

Non-biological IO services were mostly provided by cancer services; massage/touch therapies and psychological wellbeing services were the most common followed by movement modalities. Aside from much lower rates of IM consultations/advice and acupuncture, the types of IO services that were provided mostly align with international IO services [34, 37], evidence-based clinical guidelines for breast cancer and lung cancer [32, 39], and the CM therapies commonly used by cancer survivors in Australia [6]. The low rates of acupuncture services, however, were somewhat surprising. Whilst the quality of the evidence for effectiveness is variable clinical practice guidelines conclude there is moderate certainty that the net benefit from treatment is small [32, 39]. In Australia, credentialing of acupuncturists should be relatively straightforward as all acupuncturists (be they medical doctors or Chinese medicine practitioners) are statutorily regulated through the Australian Health Practitioner Regulation Agency.

Perhaps the largest mismatch was the high rates of biological-based therapies used by cancer survivors (e.g. herbs and nutritional supplements, and consultations with traditional medicine practitioners) [6, 9–11] compared to the negligible provision of biological-based IO services. Botanicals and supplements continue to be controversial due to concerns over safety, especially regarding interactions with pharmaceuticals and contraindications [40]. Decision making in this context is complex. Oncologists consistently identify a lack of knowledge and education as major barriers to discussing CM use with their patients [41–43]. An analysis of over 2000 IO consultations in a comprehensive cancer centre in the US found the most common reasons why cancer survivors sought an IO consultation with a medical doctor was to pursue a holistic integrative approach (34%), and/or to obtain expert advice on CM product use (34%) and nutrition (21%) [38]. Cancer survivors may, therefore, benefit from building positive therapeutic alliances with medical doctors and pharmacists who have specific IO training to guide the safe and effective use of CM products [38]. Little is known about the IO capacity of medical practitioners who work in cancer care in Australia. In Canada almost 70% of 100 health care providers surveyed reported that they felt unprepared to monitor cancer patients’ CM use, and fewer than 9% of participants reported being capable of searching for credible evidence-based information on CM and cancer [44]. In a survey of 176 Australian health care providers caring for patients with haematological cancer patients, 91% supported the use of mind/body therapies and 41% the use of natural products, only 19% felt they could advise patients, and 77% wanted to learn more [45, 46].

Limitations of this study include the under-representation of specialist oncologists and haematologists completing the survey. This may have biased the results that found insufficient evidence was much less important than financial and logistical barriers to providing IO. Although the acceptance of CM by Australian oncologists appears to be increasing [47], more information is needed about their views on providing IO services. It is also possible that the question enquiring whether the organisation provided IO services was answered incorrectly with some crossover regarding what was considered a non-IO service. For some respondents, CM services that were provided within existing conventional allied health services (with no additional CM practitioners) were considered non-IO, whereas others thought this was inclusive of IO. For example, physiotherapy was often associated with movement modalities. To mitigate this risk, clarification was provided to all participants during data collection, survey responses were carefully reviewed, and relevant participants were contacted to clarify their responses. Lower response rates in Northern Territory and the Australian Capital Territory limit the generalisability to these states. Despite these limitations, however, the high overall response rate and coverage of the targeted sample supports the validity and generalisability of the findings.

## Conclusion

In summary, healthcare organisations across Australia are increasingly providing IO services. Service provision, however, appears to remain limited or non-existent in many areas. Healthcare organisations signalled a need for more funding, and assistance with clinical governance and business models. Building and translating the evidence of CM and developing clinical guidelines is, therefore, suggested to inform the decisions made by clinicians, patients, and policy makers. Greater top-down strategic planning and policy guidance is indicated to ensure the appropriate provision of, and equitable access to, IO services for all cancer survivors living across Australia.

## Additional file

Additional file 1: Survey Questionnaire. (PDF 530 kb)

## Abbreviations

CM: Complementary medicine
IM: Integrative medicine
IO: Integrative oncology
