# Supplementary material for: Integrative oncology and complementary medicine cancer services in Australia: findings from a national cross-sectional survey
Source: BMC Complement Altern Med. 2018 Oct 29;18:289. doi: 10.1186/s12906-018-2357-8 (PMC6206936; doi:10.1186/s12906-018-2357-8)
Supplement: Supplementary file 1 — Survey Questionnaire. (PDF 530 kb) [file 12906_2018_2357_MOESM1_ESM.pdf]

A national survey to map Complementary Medicine service provision by Oncology Health Services across Australia

Thank you for participating in this survey. The purpose is to map hospital and community cancer services across Australia. Given the high use of Complementary Medicine by cancer survivors, the survey includes questions about these supportive cancer services. The aim is to identify gaps and barriers to service provision.

Who should complete this survey? ANY public, private or not-for-profit organisation who provides a dedicated cancer services (in-patient or out-patient). Even if your service does not offer any Complementary Medicine services, this is still important information.

Are there any benefits for you in taking part? Taking part in this survey will help build the most comprehensive overview of cancer services in Australia. We will disseminate our findings to assist both state and national cancer service planning.

Is the information provided confidential? Yes, all data will be de-identified.

How long will it take? Between 5 - 20 minutes depending on your responses (the survey has skip questions)

For further information please contact Western Sydney University
Professor Caroline Smith - caroline.smith@westernsydney.edu.au
Dr Jennifer Hunter - jennifer.hunter@westernsydney.edu.au

This research is being conducted by The National Institute of Complementary Medicine, based at Western Sydney University (WSU) and has been approved by the WSU Human Research Ethics Committee (Approval number: H11389)

We thank you for your assistance

1. General information

This will be used to track responses and clarify any questions.
All information is **confidential** and will be **de-identified**
*** Required fields**

Organisation or hospital name: *

Cancer service name (if different from above):

Postcode: *

Email address:

2. Which would accurately describe your position in the organisation? (Select ALL that apply)

- ☐ I am in administration / management
- ☐ I am a health care professional
- ☐ Other (please specify):

3. What type of organisation owns the oncology service? (Select ONE only)

- ☐ Government
- ☐ Limited company
- ☐ Not-for-profit company
- ☐ Registered charity
- ☐ Small business
- ☐ D o n ' t k n o w
- ☐ Other (please specify:

4. Which of the following cancer services are provided? (Select ALL that apply)

- ☐ Chemotherapy
- ☐ Radiotherapy
- ☐ Surgery
- ☐ Survivorship clinics
- ☐ Supportive care and allied health
- ☐ Palliative cancer care
- ☐ Wellness
- ☐ Other settings (please specify):

Comments:

5. Where are these cancer services provided? (Select ALL that apply)

- ☐ Hospital setting: in-patient beds
- ☐ Hospital setting: out-patient clinic or other room
- ☐ Community setting: clinic or centre
- ☐ Home visits / residential care visits
- ☐ Other settings (please specify):

Comments:

6. Please list any important service gaps in cancer care in your district / region

Most important unmet need:

Important unmet needs:

Comments:

The following definitions are provided for your information

Complementary Medicine – any traditional or natural therapy / practice (e.g. Oncology massage, Acupuncture, Naturopathy, Chiropractic, Osteopathy, Therapeutic touch, Reiki, Aromatherapy, Meditation, Relaxation, Yoga, Tai Chi, Music or Art therapy)

Integrative Medicine – practitioners who combine evidence-based conventional medicine with Complementary Medicine

7. Does your cancer service offer any Complementary Medicine or Integrative Medicine services? (Select ONE only)

- ☐ Yes
- ☐ No, not anymore **[GO TO Q41]**
- ☐ No, we never have **[GO TO Q41]**
- ☐ Don't know **[GO TO Q41]**
- ☐ Other (e.g. planning to provide) Please specify then **[GO TO Q41]**

Comments:

8. Where are these Complementary Medicine or Integrative Medicine services provided? (Select ALL that apply)

- ☐ Hospital setting: in-patient beds
- ☐ Hospital setting: alongside other out-patient services
- ☐ Hospital setting: a dedicated centre or clinic
- ☐ Community setting: a dedicated centre or clinic
- ☐ Community setting: not operated by our organisation
- ☐ Home visits / residential care visits
- ☐ Other settings (please specify):

Comments:

9. Approximately how long has your cancer service been providing Complementary Medicine services?

Years: Months:

Comments:

We would like to ask you some questions about the different types of Complementary Medicine and/or Integrative Medicine your service provides

10. Does your organisation offer massage that is provided by a certified Oncology Massage Therapist?

- ☐ No **[GO TO Q13]**
- ☐ We provide massage,
  but unsure whether the therapists are certified for Oncology Massage **[GO TO Q13]**
- ☐ Yes

11. If yes, then who funds the certified Oncology Massage service? (Select ALL that apply)

- ☐ Don't know
- ☐ Patient (includes rebates to patient from private health insurance or Medicare)
- ☐ Organisation / hospital
- ☐ Donations / fund raising
- ☐ Volunteers provide a free service
- ☐ Other (please specify):

Comments:

12. For the services provided by a certified Oncology Massage therapist

Number of therapists:

Total hours the services are available per week:

Comments:

---

13. OTHER MASSAGE OR TOUCH THERAPY

- ☐ We do not currently provide any other type of Massage or Touch therapy **[GO TO Q16]**
- ☐ Massage therapy (any style)
- ☐ Reflexology
- ☐ Aromatherapy
- ☐ Therapeutic touch / Reiki
- ☐ If other style of Massage is offered please list):

14. If yes, then who funds the other Massage or Touch Therapy service? (Select ALL that apply)

- ☐ Don't know
- ☐ Patient (includes rebates to patient from private health insurance or Medicare)
- ☐ Organisation / hospital
- ☐ Donations / fund raising
- ☐ Volunteers provide a free service
- ☐ Other (please specify):

Comments:

15. For the services provided by other Massage or Touch Therapists

Number of therapists:

Total hours the services are available per week:

Comments:

- -

16. BODY ALIGNMENT THERAPY

☐ We do not currently provide any type of Body Alignment therapy **[GO TO Q19]**
☐ Chiropractic
☐ Osteopathy
☐ Cranio-sacral
☐ If a different therapy is offered, please list:

17. If yes, then who funds the Body Alignment Therapy service? (Select ALL that apply)

☐ Don't know
☐ Patient (includes rebates to patient from private health insurance or Medicare)
☐ Organisation / hospital
☐ Donations / fund raising
☐ Volunteers provide a free service
☐ Other (please specify):

Comments:

18. For the services provided by Body Alignment Practitioners

Number of therapists:

Total hours the services are available per week:

Comments:

- -

19. EXERCISE AND MOVEMENT THERAPY

☐ We do not currently provide any type of Exercise or Movement therapy **[GO TO Q22]**
☐ Yoga
☐ Tai Chi
☐ Qigong
☐ Dance or Movement
☐ If a different therapy is offered, please list:

20. <u>If yes</u>, then who funds the Exercise and Movement Therapy service? (Select ALL that apply)

- ☐ Don't know
- ☐ Patient (includes rebates to patient from private health insurance or Medicare)
- ☐ Organisation / hospital
- ☐ Donations / fund raising
- ☐ Volunteers provide a free service
- ☐ Other (please specify):

Comments:

21. For the services provided by Exercise and Movement Practitioners

Number of therapists:

Total hours the services are available per week:

Comments:

- -

22. ACUPUNCTURE

- ☐ We currently do not provide any Acupuncture services **[GO TO Q25]**
- ☐ Yes, we offer Acupuncture services

23. <u>If yes</u>, then who funds the Acupuncture service? (Select ALL that apply)

- ☐ Don't know
- ☐ Patient (includes rebates to patient from private health insurance or Medicare)
- ☐ Organisation / hospital
- ☐ Donations / fund raising
- ☐ Volunteers provide a free service
- ☐ Other (please specify):

Comments:

24. For the services provided by Acupuncture Practitioners

Number of therapists:

Total hours the services are available per week:

Comments:

- -

25. INTEGRATIVE MEDICINE *(Defined as conventional medicine combined with evidence-based Complementary Medicine)*

- ☐ We do not currently provide any Integrative Medicine services **[GO TO Q28]**
- ☐ Integrative Medicine consultation
  (e.g. a medical doctor who may recommend or prescribe complementary medicines)
- ☐ Integrative Medicine advice (e.g. pharmacist advice about drug interactions)
- ☐ If another Integrative Medicine service is offered, please list:

26. If yes, then who funds the Integrative Medicine service? (Select ALL that apply)

- ☐ Don't know
- ☐ Patient (includes rebates to patient from private health insurance or Medicare)
- ☐ Organisation / hospital
- ☐ Donations / fund raising
- ☐ Volunteers provide a free service
- ☐ Other (please specify):

Comments:

27. For the services provided by Integrative Medicine Practitioners

Number of therapists:

Total hours the services are available per week:

Comments:

- -

28. MENTAL WELLBEING
(Aside from general psychology or mental health services)

- ☐ We do not currently provide any of the following Mental Wellbeing services **[GO TO Q31]**
- ☐ Relaxation
- ☐ Meditation
- ☐ Music therapy
- ☐ Art therapy
- ☐ If a different service is offered, please list:

29. If yes, then who funds the Mental Wellbeing service? (Select ALL that apply)

- ☐ Don't know
- ☐ Patient (includes rebates to patient from private health insurance or Medicare)
- ☐ Organisation / hospital
- ☐ Donations / fund raising
- ☐ Volunteers provide a free service
- ☐ Other (please specify):

Comments:

30. For the services provided by Mental Wellbeing Practitioners

Number of therapists:

Total hours the services are available per week:

Comments:

- -

31. OTHER COMPLEMENTARY MEDICINE SERVICES

- ☐ We do not currently provide any other Complementary Medicine services **[GO TO Q34]**
- ☐ Chinese herbal medicine
- ☐ Ayurvedic medicine
- ☐ Naturopathy
- ☐ Indigenous Australian medicine
- ☐ Nutritional medicine (not a dietitian service)
- ☐ If a different service is offered, please list:

32. <u>If yes</u>, then who funds the other Complementary Medicine service? (Select ALL that apply)

- ☐ Don't know
- ☐ Patient (includes rebates to patient from private health insurance or Medicare)
- ☐ Organisation / hospital
- ☐ Donations / fund raising
- ☐ Volunteers provide a free service
- ☐ Other (please specify):

Comments:

33. For the services provided by other Complementary Medicine Practitioners

Number of therapists:

Total hours the services are available per week:

Comments:

The next few questions apply to ALL the Complementary Medicine Practitioners who provide services for your organisation

34. Do your Complementary Medicine practitioners need to meet any of the following requirements?

Select ONE for each question	Must have	Preferred	Optional	No	Don't Know
Criminal record check or Working with children check	☐	☐	☐	☐	☐
First-aid certificate	☐	☐	☐	☐	☐
Credentials confirmed by your organisation	☐	☐	☐	☐	☐
Accredited by a professional association	☐	☐	☐	☐	☐
AHPRA registration *(e.g. doctor, nurse, dietitian, psychologist, Chinese medicine, osteopath, chiropractor)*	☐	☐	☐	☐	☐
Professional indemnity insurance	☐	☐	☐	☐	☐
Formal training about the organisation's procedures and protocols	☐	☐	☐	☐	☐

Comments:

35. Do any of your Complementary Medicine practitioners have dual qualifications as a biomedical trained practitioner? (e.g. Massage Therapist is also a Physiotherapist; Yoga instructor is also an Exercise Physiologist; Acupuncturist is also a Doctor; Nutritional Therapist is also a Dietitian)

☐ Don't know
☐ No
☐ Yes, please provide details:

36. Do your Complementary Medicine practitioners participate in multidisciplinary team meetings or case conferences? (Select ONE only)

☐ Yes
☐ No

Comments:

37. How are Complementary Medicine practitioner services documented? (Select ALL that apply)

- ☐ Don’t know
- ☐ A shared online clinical record within the organisation
- ☐ A shared paper-based clinical record within the organisation
- ☐ A separate record that is not integrated with the patient's clinical records but kept on-site
- ☐ A separate record that the Complementary Medicine practitioner maintains and owns
- ☐ Other (please specify):

Comments:

38. Who can use the Complementary Medicine service? (Select ALL that apply)

- ☐ All cancer patients
- ☐ Some cancer patients (please specify below)
- ☐ Family members and carers of cancer patients
- ☐ Staff
- ☐ General public
- ☐ Other (please specify):

Comments:

39. Do patients need a referral to access the Complementary Medicine services? (Select ALL that apply)

Select ONE for each question	ALL services	SOME services
NO referral is required, self-referrals are accepted	☐	☐
YES internal referral from within the organisation	☐	☐
YES referral from an oncologist	☐	☐
YES referral from any medical doctor	☐	☐
YES referral from any healthcare practitioner	☐	☐
Don’t know	☐	☐

Comments:

40. Which of the following methods are used to evaluate your Complementary Medicine services? (Select ALL that apply)

- ☐ Don't know
- ☐ None, we do not formally evaluate our services
- ☐ Patient survey
- ☐ Written patient feedback form
- ☐ Practitioner or organisation initiated clinical audit
- ☐ Observational studies
- ☐ Clinical trials
- ☐ Other (please specify):

Comments:

NOW GO TO Q43

QUESTIONS 41 & 42
ONLY FOR ORGANISATIONS WHO *DO NOT* PROVIDE ANY COMPLEMENTARY MEDICINE SERVICES

---

41. What are the reasons for not providing Complementary Medicine services? (Select ALL that apply)

- ☐ Organisational policy does not support or allow Complementary Medicine use
- ☐ No interest or support from oncologists
- ☐ Management or Board directive not wanting Complementary Medicine services
- ☐ Lack of funding
- ☐ No patient demand or awareness
- ☐ Unsure how to set up a Complementary Medicine service
- ☐ Not enough evidence to support the use of Complementary Medicine
- ☐ Unsure about which Complementary Medicine therapies to provide
- ☐ Clinical trials
- ☐ Other (please specify):

Comments:

42. What solutions can you identify to help reduce some of these barriers?

---

*This section is for **ALL SERVICES***
We would like to know about your <u>organisation's general policies</u> for Complementary Medicine

43. Does your organisation have the following in place?

	Yes	No	Decision is made on a case-by-case basis	Not applicable this is never allowed	Don't know
Credentialing policy for visiting Complementary Medicine practitioners	☐	☐	☐	☐	☐
Scope of practice for visiting Complementary Medicine practitioners	☐	☐	☐	☐	☐
Policy for referrals to Complementary Medicine practitioners outside the organisation	☐	☐	☐	☐	☐
Policy on patient-initiated herbs, vitamins and minerals	☐	☐	☐	☐	☐
Complementary Medicine information available for patients	☐	☐	☐	☐	☐

44. How are herbs, vitamins and minerals documented in the patient's clinical record? (Select ONE only)

- ☐ N/A patients are not permitted to use these products
- ☐ ALL products (including self-initiated) are listed on the medication chart
- ☐ ONLY products approved by medical staff are listed on the medication chart all other product use is documented in the clinical history
- ☐ Product use is ONLY documented in the clinical history
- ☐ Don't know
- ☐ Other (please specify):

Comments:

45. Have you heard of The Council of Australian Therapeutic Advisory Groups (CATAG)?

- ☐ Yes
- ☐ No **[GO TO Q47]**
- ☐ N/A we are not a hospital organisation **[GO TO Q47]**

46. If yes, then thinking about your organisation and CATAG's Position Statement for the use of Complementary and Alternative Medicines, 2015

http://www.catag.org.au/wp-content/uploads/2012/08/150518_CAM-Position-statement-final.pdf

Our policies are aligned with this CATAG statement

- ☐ Yes
- ☐ No

Comments:

The last few questions are about Oncology Massage

47. In your opinion, who is likely to deliver safe massage to cancer patients / survivors?

	Very likely	Likely	Possibly	Unlikely	Very unlikely	Don't know
Family member or friend	☐	☐	☐	☐	☐	☐
Volunteer	☐	☐	☐	☐	☐	☐
Health Practitioner (no massage training)	☐	☐	☐	☐	☐	☐
Massage Therapist (any style)	☐	☐	☐	☐	☐	☐
Certified Oncology Massage Therapist	☐	☐	☐	☐	☐	☐

Comments:

48. Is your cancer service aware of any evidence demonstrating that the training of the massage practitioner can influence patient outcomes?

- ☐ Yes
- ☐ No
- ☐ Don't know

Comments:

49. Is your cancer service aware of any evidence that Oncology Massage can reduce pain or anxiety in cancer patients?

- ☐ Yes
- ☐ No
- ☐ Don't know

Comments:

50. Are there any barriers to providing Oncology Massage in your organisation?
(i.e. massage provided by a certified Oncology Massage Therapist)

- ☐ Yes
- ☐ No **[GO TO Q52]**
- ☐ Don't know **[GO TO Q52]**

Comments:

51. If yes, what are the barriers? (Select ALL that apply)

- ☐ Doctors or allied health do not support Oncology Massage
- ☐ Funding not available for Oncology Massage
- ☐ Unable to recruit certified Oncology Massage Therapists
- ☐ Low patient demand
- ☐ Don't know
- ☐ Other (please specify):

Comments:

---

52. Do you have any final comments?
(e.g. patient needs, challenges, lessons learnt, future plans for services)

Comments:

53. Who can we contact if we need to clarify any questions?
Name:
Email address:
Phone number:

54. To receive a copy of the results please provide postal or email details (if different to above)
Name:
Organisation:
Address:

City/Town:
State: Postcode: '
Email address:

Thank you for participating in this survey
